# Choroidal vasculature act as predictive biomarkers of long-term ocular elongation in myopic children treated with orthokeratology: a prospective cohort study

**DOI:** 10.1186/s40662-023-00345-2

**Published:** 2023-06-06

**Authors:** Hao Wu, Tianli Peng, Weihe Zhou, Zihan Huang, Hongyu Li, Tengfei Wang, Jingwei Zhang, Kou Zhang, Haoer Li, Yunpeng Zhao, Jia Qu, Fan Lu, Xiangtian Zhou, Jun Jiang

**Affiliations:** 1 National Clinical Research Center for Ocular Diseases, Eye Hospital, Wenzhou Medical University, Wenzhou, 325027 China; 2 State Key Laboratory of Ophthalmology, Optometry and Visual Science, Eye Hospital, Wenzhou Medical University, Wenzhou, 325027 China; 3 Research Unit of Myopia Basic Research and Clinical Prevention and Control, Chinese Academy of Medical Sciences (2019RU025), Wenzhou, Zhejiang China

**Keywords:** Myopia control, Orthokeratology, Choroidal vasculature, Biomarker, Prediction

## Abstract

**Background:**

Despite receiving orthokeratology (ortho-k), the efficacy of retarding ocular elongation during myopia varies among myopic children. The current study aimed to investigate the early changes of choroidal vasculature at one month after ortho-k treatment and its association with one-year ocular elongation, as well as the role of such choroidal responses in predicting the one-year control efficacy of ortho-k treatment.

**Methods:**

A prospective cohort study was conducted in myopic children treated with ortho-k. Myopic children aged between 8 and 12 years who were willing to wear ortho-k lenses were recruited consecutively from the Eye Hospital of Wenzhou Medical University. Subfoveal choroidal thickness (SFCT), submacular total choroidal luminal area (LA), stromal area (SA), choroidal vascularity index (CVI), choriocapillaris flow deficit (CcFD) were evaluated by optical coherence tomography (OCT) and OCT angiography over a one-year period.

**Results:**

Fifty eyes from 50 participants (24 males) who finished one-year follow-ups as scheduled were included, with a mean age of 10.31 ± 1.45 years. The one-year ocular elongation was 0.19 ± 0.17 mm. The LA (0.03 ± 0.07 mm^2^), SA (0.02 ± 0.05 mm^2^) increased proportionally after one-month of ortho-k wear (both *P* < 0.01), as did the SFCT (10.62 ± 19.98 μm, *P* < 0.001). Multivariable linear regression analyses showed that baseline CVI (β = − 0.023 mm/1%, 95% CI: − 0.036 to − 0.010), one-month LA change (β = − 0.009 mm/0.01 mm^2^, 95% CI: − 0.014 to − 0.003), one-month SFCT change (β = − 0.035 mm/10 µm, 95% CI: − 0.053 to − 0.017) were independently associated with one-year ocular elongation during ortho-k treatment after adjusting with age and sex (all *P* < 0.01). The area under the receiver operating characteristic curve of prediction model including baseline CVI, one-month SFCT change, age, and sex achieved 0.872 (95% CI: 0.771 to 0.973) for discriminating children with slow or fast ocular elongation.

**Conclusions:**

Choroidal vasculature is associated with ocular elongation during ortho-k treatment. Ortho-k treatment induces increases in choroidal vascularity and choroidal thickness as early as one month. Such early changes can act as predictive biomarkers of myopia control efficacy over a long term. The utilization of these biomarkers may help clinicians identify children who can benefit from ortho-k treatment, and thus has critical implications for the management strategies towards myopia control.

**Supplementary Information:**

The online version contains supplementary material available at 10.1186/s40662-023-00345-2.

## Background

Myopia is a common condition that develops primarily during childhood when excessive ocular elongation results in images of distant objects focusing in front of the retina [1, 2]. It has already affected nearly 34% of the global population, which is anticipated to become 50% by 2050 [3]. The increase in axial length (AL) during myopia development greatly enhances the risks of developing vision-threatening ocular complications in later life [4, 5]. Therefore, it is critical to slow the early progression of myopia with adequate intervention.

Orthokeratology (ortho-k) is one of the most widely used optical interventions for myopia control [6]. It retards ocular elongation by 32% to 55% per year by reshaping corneal topography [7], which consequently induces changes in optical signals received by the retina, but the mechanism underlying this process remains elusive. However, about 27% of children receiving ortho-k treatment, for over one year, still progressed by at least 1.00 D of myopia and had increases of 0.40 mm in ocular size [8]. Since the estimation of treatment efficacy usually requires a one-year timeframe, it would hamper the timely commencement of effective myopia control measures, and thus inevitably increasing the possibility of myopia progression.

To address this issue, researchers have identified various factors that affect the rate of ocular elongation with ortho-k treatment, such as age and myopic refraction at the start of treatment, and changes in corneal topography after ortho-k wear [9]. Besides these, Lau et al. observed choroidal thickening as early as one week after the ortho-k wear [10]. Another study showed that an initial increase in choroidal thickness after one month of ortho-k treatment was associated with less ocular elongation over a one-year period of continued ortho-k wear [11], but the role of choroidal thickness and its discriminative ability for myopia control efficacy are yet to be illustrated.

Compelling evidence show that alteration in choroidal thickness is an early sign of vision-driven changes in ocular growth and myopia development [12, 13], wherein choroidal thickness decreases during the development of experimental myopia and increases in response to imposed myopic defocus or upon the removal of myopiagenic stimuli that basically introduces a myopic defocus [14–16]. In line with choroidal thickness, choroidal blood flow also exhibits a bidirectional response to visual stimuli [17], which may act through mitigating scleral hypoxia and remodeling [18–20]. Recent studies on animal models of myopia also showed that the inhibitory effect of several anti-myopia treatments (atropine, apomorphine and intense light) was associated with the improvement of choroidal blood flow [19]. These studies raise the possibility that choroidal blood flow act as a mediator of ortho-k driven myopia control.

The objective of the current study was to investigate the early changes of choroidal vasculature at one month after ortho-k treatment and its association with one-year ocular elongation, as well as the role of such choroidal responses in predicting the one-year control efficacy of ortho-k treatment. Myopic children treated with ortho-k were recruited in this prospective study. Other than choroidal thickness, a detailed assessment of choroidal vasculature with choroidal vascularity and choriocapillaris flow deficits was also performed with the optical coherence tomography (OCT) system. Findings from this study would give insights into optimizing treatment strategies for early intervention of myopia control.

## Methods

### Subjects

This prospective cohort study was conducted at Eye Hospital of Wenzhou Medical University (Wenzhou, China). The study was conducted in accordance with the tenets of the Declaration of Helsinki and was approved by the ethical committee of Eye Hospital of Wenzhou Medical University. Written informed consent was obtained from all participants and their parents.

Subjects were recruited and screened consecutively between August 2020 and September 2021. Children who were willing to wear ortho-k lenses were enrolled and followed up for one year. The inclusion criteria were as follows: aged between 8 and 12 years, corrected visual acuity of 20/20 or better, mean spherical equivalent refraction (SER) between − 5.00 D and − 0.75 D, astigmatism no greater than 2.00 D, interocular difference in SER of less than or equal to 1.50 D. The exclusions were those with active inflammatory or ocular surface diseases, strabismus, history of using myopia control modalities such as rigid contact lenses, multifocal soft contact lenses, and atropine.

The participants were advised to wear their ortho-k lenses every night for at least 8 h and ocular examinations were performed at the start of ortho-k treatment, and 1 day, 1 week, 1 month, 3 months, 6 months, 9 months, and 12 months after ortho-k wear. At each visit, unaided visual acuity, manifest refraction (if unaided visual acuity was worse than 20/25), and corneal topography (Medmont E-300, Australia) were assessed. Slit-lamp examination was also conducted to check for lens fitting and contact lens related complications. AL using the IOL-Master 700 (Carl Zeiss Meditec AG, Jena, Germany) and choroidal parameters using swept-source OCT/OCT angiography (SS-OCT/OCTA) were measured at baseline, 1 month, 6 months and 12 months. All examinations were performed at the same time of the day (in the morning or at afternoon) at each visit to minimize the diurnal changes.

### SS-OCT/OCTA scanning and analysis

The SS-OCT/OCTA system (VG200S; SVision Imaging, Henan, China) contained a swept-source laser with a central wavelength of approximately 1050 nm and a scan rate of 200,000 A-scans per second. The system was equipped with an eye tracking utility based on an integrated confocal scanning laser ophthalmoscope to eliminate eye-motion artifacts. The axial resolution was 5 μm, lateral resolution was 13 μm, and scan depth was 3 mm.

Structural OCT of the macular region was performed with 18 radial scan lines centered on the fovea. Each scan line, generated by 2048 A-scans, was nominally 12 mm long and separated from the adjacent lines by 10°. Sixty-four B-scans were obtained on each scan line and were automatedly averaged to improve the signal-to-noise ratio. Only the horizontal scans across the fovea and optic disc were representatively used to analyze the choroidal thickness and choroidal vascularity (refer to Additional file 1a for details). The choroid in the SS-OCT images was defined as the area between the retinal pigment epithelium (RPE)-Bruch’s membrane complex and the choroid-sclera interface. The segmentation was defined automatically with a custom-built Python package, then the image was binarized to differentiate the choroidal luminal area (LA) and stromal area (SA) using a custom algorithm developed in MATLAB R2018b (MathWorks, Natick MA, USA) (Additional file 1b and c) [21, 22]. After image processing, LA, SA, total choroidal area (TCA, calculated as the sum of LA and SA), choroidal vascularity index (CVI, defined as the ratio of LA to TCA), subfoveal choroidal thickness (SFCT) were measured. The 6-mm macular region centered on the fovea was regarded as the region of interest (Additional file 1d).

For angiography, the 3-dimensional volumetric data were obtained with a raster scan protocol of 512 continuous horizontal B-scans that covered an area of 6 mm × 6 mm centered on the fovea. A supplementary figure file shows this in more detail (see Additional file 2a). Each B-scan contained 512 A-scans and was repeated four times and averaged. Enface angiograms of the choriocapillaris slab were evaluated, which was defined by a layer starting at the basal border of the RPE-Bruch’s membrane complex and ending at approximately 20 μm beneath the RPE-Bruch’s membrane complex (Additional file 2b). Choriocapillaris flow deficits (CcFDs) were defined as regions having no flow signals that were detectable by the threshold binarization algorithm (Additional file 2c), as previously described [23]. The CcFD percentage (CcFD%) was calculated by dividing the area of the CcFDs by the area of the measured region and then converting the values to percentages. Limited by image resolution at the scan edges, only the 5 mm diameter circular region centered on the fovea was used for analysis (Additional file 2c).

The scales of both OCT and OCTA images were adjusted for the differences in magnification due to differences in AL among the individuals[24]. The choroidal measurements exhibited good repeatability and reproducibility, as previously reported [21, 22, 25, 26].

### Statistics

Participants with complete data at the start of ortho-k wear, and one month and one year post-treatment were included in the final analyses. Due to the symmetry of both eyes from the individuals, data from the right eyes were arbitrarily used for analysis. To minimize the influence of the window period, one-year AL elongation was adjusted by the number of days of ortho-k wear.

Statistical analyses were performed using SPSS Statistics (version 26.0, IBM, Armonk, NY, USA) and R version 4.2.0 (R Foundation for Statistical Computing). The normality of data was examined by the Shapiro-Wilk test. Descriptive data were presented using means and standard deviations or number and proportion, and compared using a t-test, Mann-Whitney test, or Chi-squared test where appropriate. Pearson’s or Spearman’s correlation was used to calculate the degree and statistical significance of associations between variables wherever appropriate. The linear regression model was used to assess factors associated with one-year AL elongation. Factors with *P* values less than 0.05 in univariable analyses were added into the multivariable models, where age and sex were entered as covariables in the models as well.

To assess the prediction value of choroidal biomarkers, receiver operating characteristic curve (ROC) with area under the curve (AUC) was used to quantify the predictive discrimination for children with slow or fast AL progression, which is defined according to the median level of the cohort with one-year AL elongation. Delong test was used to assess the differences between AUCs of different models. In addition, integrated discrimination improvement (IDI) with Z test was applied to assess improved prediction accuracy after addition with other predictors in previous models. Depending on the most valuable prediction model, a candidate nomogram model was eventually constructed with good predictive performance. To visualize the results, each 10 μm change in SFCT and each 0.01 mm^2^ change in LA, SA and TCA was considered a one unit change in the linear and prediction models. A two-sided *P* value of less than 0.05 was considered statistically significant.

## Results

Out of the 74 that were enrolled, a total of 50 (68%) participants (24 males/26 females) completed the follow-up visits. Table 1 shows baseline characteristics of the entire cohort, and the differences between subgroups with one-year AL elongation below or above the median level of 0.16 mm (designated as the slow progression and fast progression subgroups, respectively). The mean age was 10.31 ± 1.45 years old. The mean SER, average corneal keratometry (K) at a central 3-mm zone, and AL were −2.86 ± 1.09 D, 43.43 ± 1.29 D, and 24.77 ± 0.73 mm at baseline, respectively. The mean AL elongation over one year of ortho-k wear was 0.19 ± 0.17 mm across all individuals, with an elongation of 0.06 ± 0.09 mm in the slow progression subgroup and 0.32 ± 0.12 mm in the fast progression subgroup. Participants with slow progression were significantly older than those with fast progression (10.72 ± 1.47 years vs. 9.91 ± 1.34 years, *P* = 0.047). The CVI at baseline was significantly higher in the slow progression subgroup than in the fast progression subgroup. There were no significant differences in other ocular and choroidal metrics at baseline between these two progression subgroups (Table 1).

**Table 1:** Baseline characteristics of the cohort

Characteristics	Total (n = 50)	Progression ≤ 0.16 mm (n = 25)	Progression > 0.16 mm (n = 25)	*P* value
Demographics				
Age (years)	10.31 ± 1.45	10.72 ± 1.47	9.91 ± 1.34	**0.047**^a^
Sex (male/female)	24/26	15/10	9/16	0.089^b^
Ocular biometrics				
SER (D)	− 2.86 ± 1.09	− 2.84 ± 1.16	− 2.87 ± 1.03	0.924^a^
AL (mm)	24.77 ± 0.73	24.77 ± 0.71	24.77 ± 0.76	0.992^a^
Average K (D)	43.43 ± 1.29	43.46 ± 1.10	43.41 ± 1.48	0.888^a^
Choroidal metrics				
LA (mm^2^)	0.84 ± 0.18	0.89 ± 0.19	0.80 ± 0.16	0.079^a^
SA (mm^2^)	0.55 ± 0.10	0.55 ± 0.11	0.55 ± 0.10	0.915^a^
TCA (mm^2^)	1.39 ± 0.27	1.44 ± 0.29	1.35 ± 0.24	0.264^a^
SFCT (µm)	245.41 ± 50.86	252.58 ± 56.74	238.24 ± 44.21	0.324^a^
CVI (%)	60.50 ± 3.07	61.88 ± 2.23	59.11 ± 3.21	**0.001**^c^
CcFD (%)	13.53 ± 1.76	13.64 ± 1.92	13.42 ± 1.61	0.660^a^

*SER* = spherical equivalent refraction; *AL* = axial length; *K* = corneal keratometry; *LA* = luminal area; *SA* = stromal area; *TCA* = total choroidal area; *SFCT* = subfoveal choroidal thickness; *CVI* = choroidal vascularity index; *CcFD* = choriocapillaris flow deficits

^a^*P* value determined by independent t-test

^b^*P* value determined by Chi-squared test

^c^*P* value determined by Mann-Whitney test

Bold font indicates statistical significance

### One-month choroidal changes following ortho-k wear

The curvature of the central cornea became significantly flattened after one-month of ortho-k treatment (change − 1.47 ± 1.01 D, *P* < 0.001), with no significant difference between the two progression subgroups (− 1.25 ± 1.09 D in the slow progression subgroup vs. −1.69 ± 0.88 D in the fast progression subgroup, *P* = 0.065). The choroidal structure and vasculature were also altered significantly compared to baseline after one month of ortho-k treatment, with the LA, SA, TCA, SFCT increasing by 0.03 ± 0.07 mm^2^, 0.02 ± 0.05 mm^2^, 0.06 ± 0.11 mm^2^, 10.62 ± 19.98 μm, respectively (all *P* < 0.01, Additional file 3). CVI and CcFD showed no significant change at the one-month visit. The changes in choroidal structure and vasculature were significantly larger in the slow progression subgroup than those of the fast progression subgroup (all *P* < 0.05), except CVI and CcFD (Additional file 3).

### Association between one-month changes in choroid and one-year ocular elongation

**Fig. 1:**
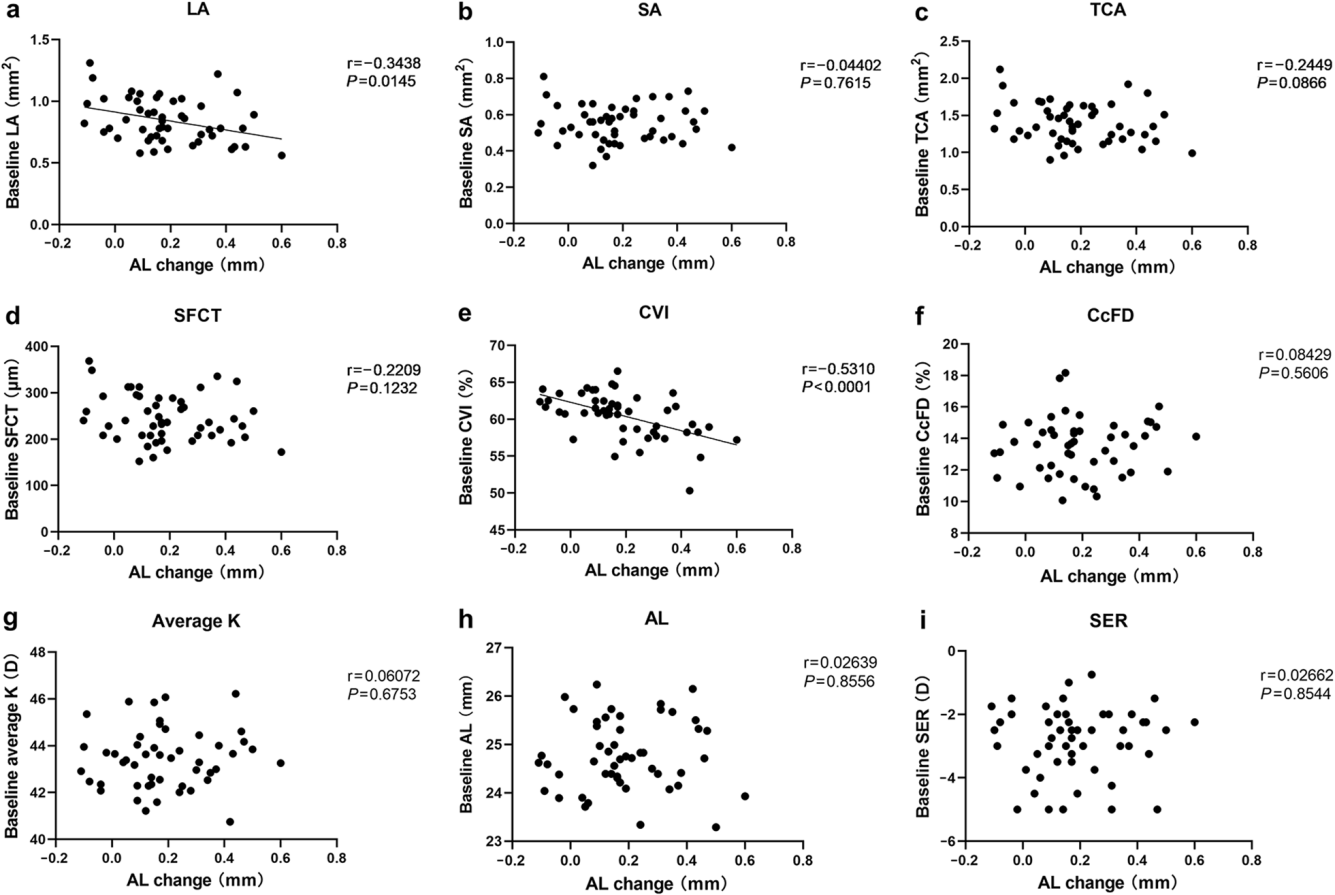
Correlation of metrics at baseline with one-year AL elongation.** a** LA; **b** SA; **c** TCA; **d** SFCT; **e** CVI; **f** CcFD; **g** Average K; **h** AL; **i** SER. Those parameters with a significant correlation with AL were fitted with a regression line. AL, axial length; LA, luminal area; SA, stromal area; TCA, total choroidal area; SFCT, subfoveal choroidal thickness; CVI, choroidal vascularity index; CcFD, choriocapillaris flow deficits; K, corneal keratometry; SER, spherical equivalent refraction

**Fig. 2:**
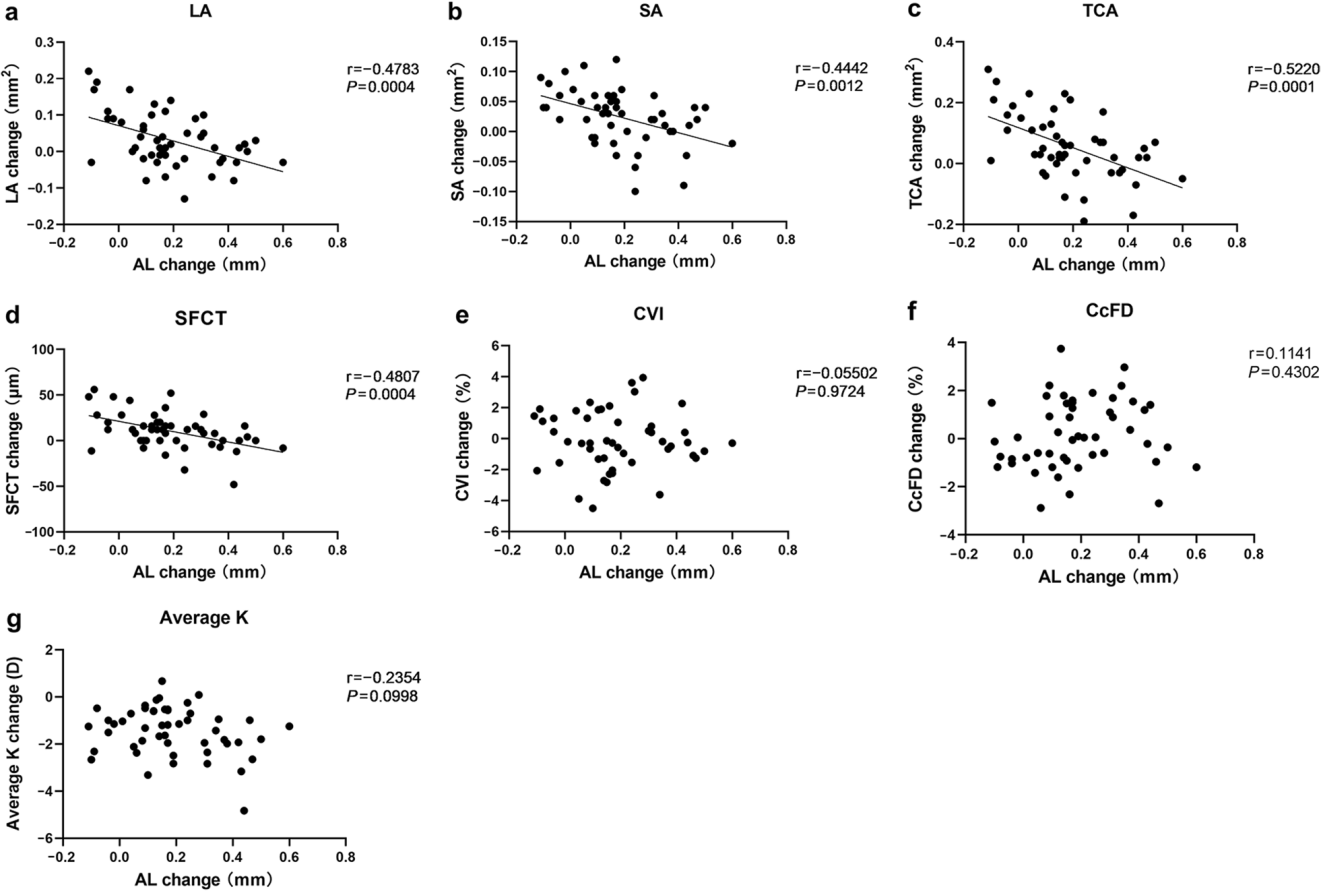
Correlation of one-month changes in corneal and choroidal metrics with one-year AL elongation.** a** LA; **b** SA; **c** TCA; **d** SFCT; **e** CVI; **f** CcFD; **g** Average K. Parameters with a significant correlation with AL were fitted with a regression line. AL: axial length; LA: luminal area; SA: stromal area; TCA: total choroidal area; SFCT: subfoveal choroidal thickness; CVI: choroidal vascularity index; CcFD: choriocapillaris flow deficits; K: corneal keratometry

Figures 1 and 2 show the scatter plots of baseline characteristics, as well as one-month changes in choroid and cornea, against the one-year AL elongation. Among these, baseline LA (r = − 0.344, *P* = 0.015), baseline CVI (r = − 0.531, *P* < 0.001), as well as one-month changes in LA (r = − 0.478, *P* < 0.001), SA (r = − 0.444, *P* = 0.001), TCA (r = − 0.522, *P* = 0.001) and SFCT (r = − 0.481, *P* < 0.001), showed a significant negative correlation with one-year AL elongation.

Correlation matrix (Additional file 4) further showed high inter-correlation among baseline LA, SA, TCA and SFCT, except CVI. There was also high inter-correlation among one-month changes in LA, TCA and SFCT. To avoid multicollinearity, two multivariable linear regression models were constructed (Table 2). Model 1 included baseline CVI, baseline LA, one-month changes in LA and SA, while model 2 included baseline CVI, baseline LA and SFCT changes. Both models were adjusted for confounding factors, i.e., age and sex. The results showed baseline CVI (β = − 0.023 mm/1%, *P* = 0.001), one-month changes in LA (β = − 0.009 mm/0.01 mm^2^, *P* = 0.004) and one-month changes in SFCT (β = − 0.035 mm/10 µm, *P* < 0.001) were independently associated with one-year AL elongation during ortho-k treatment. Therefore, these three metrics could possibly act as potential biomarkers for further prediction analyses.

**Table 2:** Choroidal metrics associated with one-year AL elongation

Parameters	Multivariable model 1		Multivariable model 2	
	β (95% CI)	*P* value	β (95% CI)	*P* value
Baseline CVI, %	− 0.023 (− 0.036 to − 0.010)	**0.001**	− 0.023 (− 0.036 to − 0.010)	**0.001**
Baseline LA, per 0.01 mm^2^	− 0.001 (− 0.003 to 0.001)	0.420	− 0.001 (− 0.003 to 0.001)	0.322
LA change, per 0.01 mm^2^	− 0.009 (− 0.014 to − 0.003)	**0.004**	–	–
SA change, per 0.01 mm^2^	− 0.003 (− 0.013 to 0.006)	0.500	–	–
SFCT change, per 10 μm	–	–	− 0.035 (− 0.053 to − 0.017)	**< 0.001**

*CVI* = choroidal vascularity index; *LA* = luminal area; *SA* = stromal area; *SFCT* = subfoveal choroidal thickness

One-month changes in LA + SA and SFCT was separately included in model 1 and model 2 due to the collinearity. The multivariable linear models were adjusted with age and sex

Bold font indicates statistical significance

### Performance of prediction models with choroidal biomarkers

**Fig. 3:**
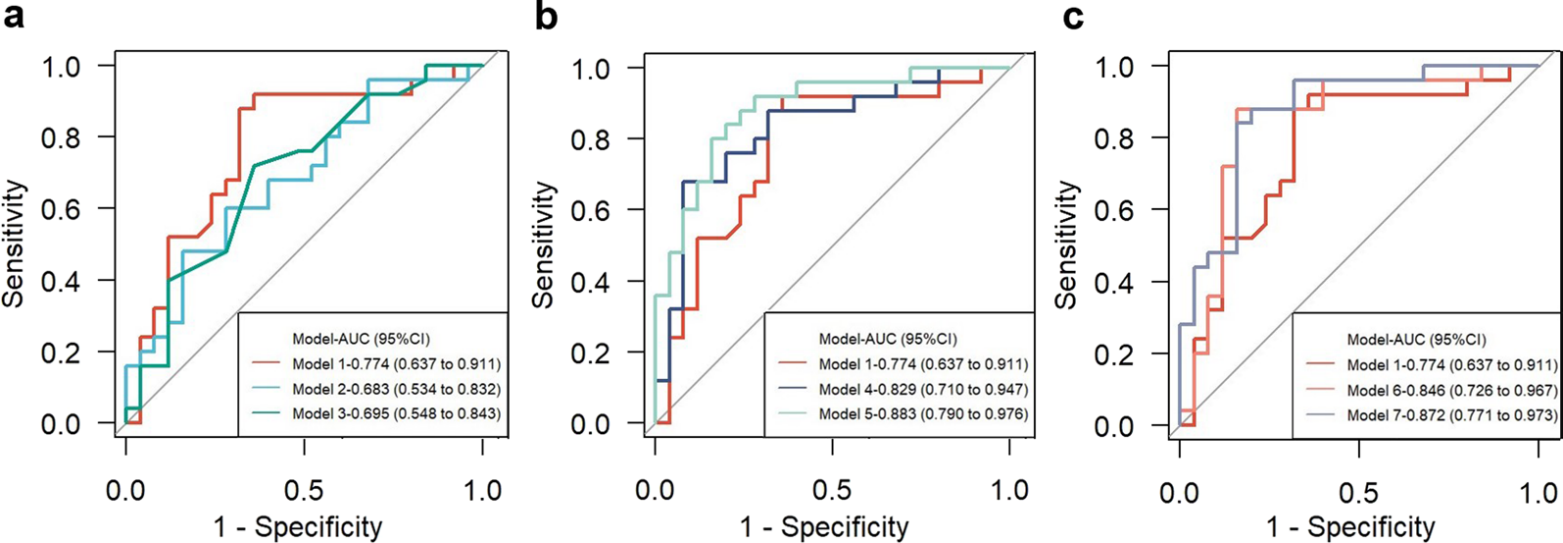
ROC of the prediction models for discriminating slow and fast AL progression with one-year orthokeratology.** a** Choroidal biomarkers alone; **b** Baseline CVI addition with LA change, baseline age and sex; **c** Baseline CVI addition with SFCT change, baseline age and sex. Model 1: baseline CVI; Model 2: LA changes; Model 3: SFCT changes; Model 4: baseline CVI + LA changes; Model 5: baseline CVI + LA changes + Age + Sex; Model 6: baseline CVI + SFCT changes; Model 7: baseline CVI + SFCT changes + Age + Sex. ROC, receiver operating characteristic curve; AL, axial length; CVI, choroidal vascularity index; LA, luminal area; SFCT, subfoveal choroidal thickness

**Fig. 4:**
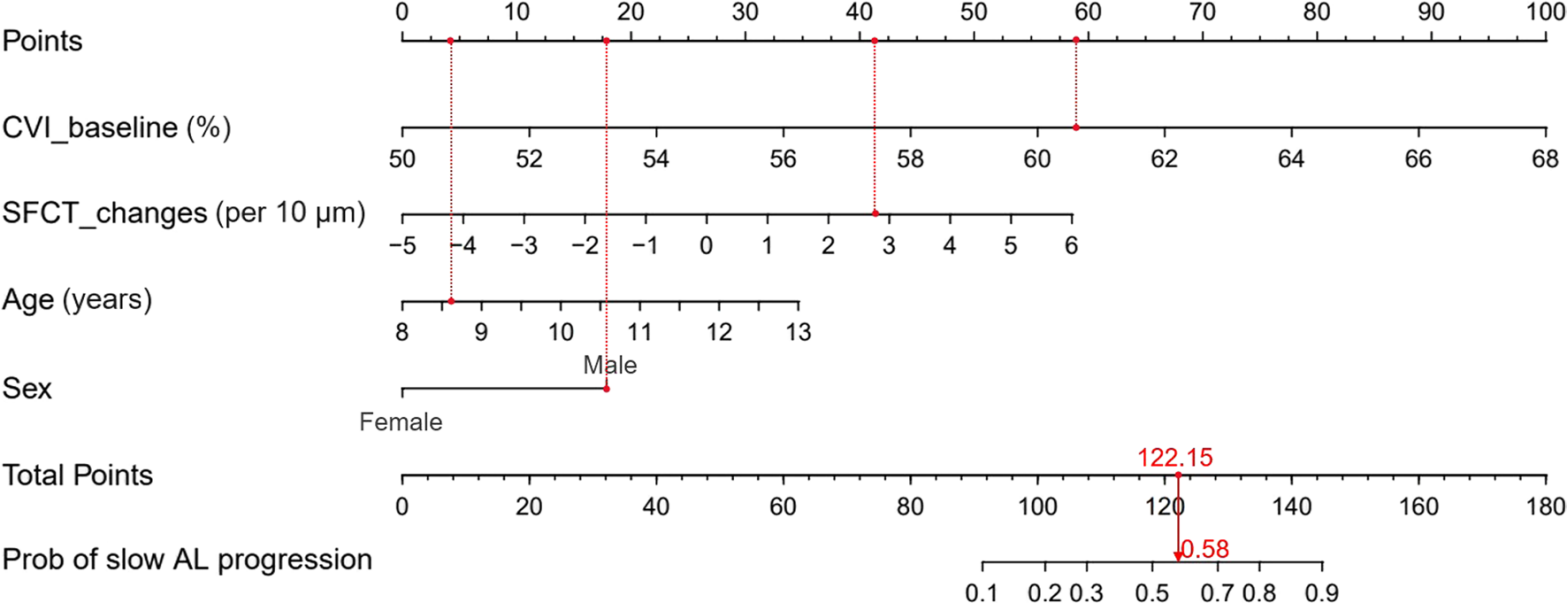
Nomogram including baseline CVI and SFCT change to predict the probability of slow AL progression. Patient 1 (male, 8 years old) from this study is shown as an example (presented in red). The baseline CVI was 60.55% and one-month SFCT change was 27.96 μm. To use the nomogram, the specific points (black dots) of the individual patient are located on each variable axis. Red lines and dots are drawn upward to determine the points received by each variable; the sum (122.15) of these points is located on the Total Points axis, and a line is drawn downward to the axes to determine the probability of slow AL progression under orthokeratology treatment (58%). CVI, choroidal vascularity index; SFCT, subfoveal choroidal thickness; AL, axial length

Figure 3 illustrates the AUCs for prediction models with choroidal biomarkers to identify ortho-k wearers with slow or fast AL progression. Models that only included baseline CVI, LA changes, or SFCT changes achieved AUCs of 0.774 (95% CI: 0.637 to 0.911), 0.683 (95% CI: 0.534 to 0.832) and 0.695 (95% CI: 0.548 to 0.843), respectively. Since there was no significant difference in AUCs among these three models, a combination of baseline CVI with LA change or SFCT change was performed to construct a prediction model. The model with baseline CVI and LA change achieved an AUC of 0.829 (0.829 vs. 0.774, *P* = 0.198) and IDI of 8.7% (95% CI: 0.3% to 17.1%, *P* = 0.042), while the model with baseline CVI and SFCT change achieved an AUC of 0.846 (0.846 vs. 0.774, *P* = 0.169) and IDI of 10.2% (95% CI: 1.8% to 18.6%, *P* = 0.017), indicating improvement of prediction accuracy. Further addition of age and sex into the above mentioned two models achieved an AUC of 0.883 (0.883 vs. 0.829, *P* = 0.250) and IDI of 13.1% (95% CI: 3.5% to 22.8%, *P* = 0.008) in model including LA change, and an AUC of 0.872 (0.872 vs. 0.846, *P* = 0.597) and IDI of 10.2% (95% CI: 1.4% to 18.9%, *P* = 0.023) in the model including SFCT change. Therefore, two final models were constructed: one including baseline CVI, LA change, age, and sex; another including baseline CVI, SFCT change, age and sex. There was no significant difference in terms of AUC and IDI between them. SFCT was measured, which is relatively easier than to measure the LA, where one needs to consider the size of the region of interest. Finally, a nomogram, which included baseline CVI, one-month SFCT change, age, and sex, was generated for predicting the probability of slow AL progression after one-year ortho-k treatment (Fig. 4). In this case, the one-year ocular elongation rate could be predicted, using the nomogram, in children wearing ortho-k lens for one month.

## Discussion

Finding the predictive biomarkers of treatment response during ortho-k wear is clinically significant in myopia control. This enables early detection and filtering of myopic individuals that are likely to benefit from this treatment. In this study, we found that the choroidal structure and vasculature were altered during the early stages of ortho-k treatment. Specifically, the indicators of choroidal vascularity (LA and SA), as well as SFCT, increased after one-month of treatment, with proportional changes as indicated by the stabilization of CVI. Notably, the baseline CVI, early increment of LA and SFCT were independently associated with ocular elongation after one-year of ortho-k treatment. Baseline CVI alone could predict one-year AL control efficacy with reasonable discrimination. Addition of age, sex, and one-month SFCT change could improve the prediction accuracy.

### Role of choroid in ortho-k-driven retardation of myopia progression

Studies involving a wide range of animal species indicate that the choroid is involved in transmitting the visual signals from the retina to the sclera that mediate ocular growth and myopia development [12]. Imposed myopic and hyperopic defocus in young animals lead to a rapid thickening and thinning of the choroid, respectively [14–16]. Accompanied by changes in choroidal thickness, choroidal blood flow also decreased during myopia development and increased upon the removal of myopiagenic stimulus [17, 27, 28]. Such choroidal changes preceded and contributed to the scleral extracellular matrix remodeling and ocular elongation that were primarily triggered by scleral hypoxia [16, 18–20].

Overnight ortho-k wear reshapes the corneal topography by flattening the central and steepening mid-peripheral zones, which induces clear unaided central vision and relative peripheral myopic defocus in the retina [29–31]. Meanwhile, increased ocular higher-order aberrations were also observed after ortho-k [32, 33]. Such alterations in optical signals contributed to increased choroidal thickness and choroidal blood flow as previously reported [34, 35], which is similar to the short-term defocus experimental paradigms conducted in young adults [36, 37]. Consequently, the sustained increases in choroidal blood flow may mitigate scleral hypoxia and scleral remodeling, ultimately retarding ocular elongation during myopia development.

Previous studies on the predictive value of baseline choroidal thickness for ocular elongation in children and in animal models have yielded inconsistent results. In a population-based cohort study, a thicker choroid at baseline was associated with increased five-year ocular elongation in children without myopia, whereas there was no correlation between baseline choroidal thickness and five-year axial elongation in children with myopia [38]. In chicks, there was no association between baseline choroidal thickness and subsequent ocular elongation during experimental myopia, whereas a negative association existed between them upon a myopia inhibitory treatment [39, 40]. However, in guinea pigs, baseline choroidal thickness was negatively associated with ocular elongation in conditions of naturally occurring and experimentally-induced myopia [41]. As highlighted by our study, a higher baseline CVI, instead of baseline SFCT, correlated with less ocular elongation during ortho-k treatment. These discrepancies may be attributed to the fact that choroidal vasculature was complex and cannot simply be represented with thickness [25, 42–44]. These findings indicate that CVI may determine the susceptibility of ocular elongation to myopia control treatment. Whether it determines the susceptibility in other conditions and the underlying mechanisms could warrant further studies.

### Predictive value of choroidal biomarkers for treatment efficacy

Overnight ortho-k achieved an effect of 32% to 55% on slowing ocular elongation during myopia development in children, with an average ocular elongation of ~ 0.16 mm per year during the first two years [7, 45]. Such ocular elongation rates are comparable to the annual physiological eye growth observed in emmetropic and myopic children without a significant myopic shift [46–49], which has been suggested to be the AL target for myopia control [50]. However, myopia control efficacy varies among ortho-k lens wearers [8, 51, 52]. Therefore, an important clinical issue that remains to be resolved is the identification of children in whom ortho-k is likely to be most effective.

Based on the involvement of choroidal blood flow in the local signaling cascades, changes in choroidal thickness during ortho-k treatment have been noted recently [13, 45]. The choroid thickened by approximately 10 to 20 μm between one week to one month and stabilized during one year of ortho-k wear [11, 34, 35, 53]. A greater initial choroidal thickening, probably attributed to the dilation of vessels [34, 35], was associated with less ocular elongation over a one-year period [11], suggesting that short-term changes in the choroid may be a biomarker for longer-term eye growth. Here, we found that, in addition to SFCT, the increases in LA after one month were also associated with decreases in ocular elongation over the one-year period.

Notably, we demonstrated the predictive value of initial choroidal responses at one month for identifying the children with slow or fast AL progression over one-year ortho-k treatment (defined as one-year ocular elongation below the median level of 0.16 mm, which is close to the annual physiological eye growth) [46–49], The results showed that compared with the crude model including baseline CVI (AUC 0.774), the final model including baseline CVI, one-month LA change, age and sex achieved an AUC of 0.883 and significantly improved the prediction accuracy in terms of IDI. Instead of one-month LA change, the model including one-month SFCT change showed a comparable prediction performance in terms of AUC and IDI. These results suggest that choroidal thickness, particularly SFCT, can be used as a surrogate biomarker of choroidal vascularity for the prediction of AL control efficacy with ortho-k treatment when considering the practicality of implementing it in a clinical setting. We expect that with the development of the OCT technique, a higher precision and convenience for the evaluation of choroidal blood flow can offer better performance of prediction.

### Strengths and limitations

This study benefits from a prospective design and detailed assessment of choroidal characteristics. Limitations in our study, such as the inability to follow up with 24 out of 74 individuals due to the COVID-19 pandemic should be acknowledged. Another limitation is the assumed equivalence between the slow and fast subgroups in the optical myopia control signal originating from the ortho-k treatment according to the changes in the central corneal curvature. The topographical changes in the mid-periphery of the cornea may also have disclosed differences that account for some of the differences in the ocular response [54, 55], In addition, environmental factors, such as time outdoors during ortho-k treatment were not accounted for. Although animal studies cannot draw a definitive conclusion of intense light (one of the most possible biological mechanism of time outdoors) on the regulation of ocular growth and refractive development disturbed by imposed hyperopic or myopic defocus [56–58], increased time outdoors has been suggested to slow the progression and onset of myopia in children [59–61]. Therefore, issues regarding corneal optics and environmental factors should be addressed when elucidating the potential biological links in future studies. Moreover, choroidal vasculature was analyzed only with horizontal scans, whereas a volumetric analysis would have provided more information. Lastly, the sample size may be too small to confidently perform prediction model analyses. A larger cohort study with external validation is needed to improve the performance of the prediction model.

## Conclusion

Choroidal vasculature is associated with ocular elongation during ortho-k treatment. Ortho-k treatment induces increases in choroidal vascularity and choroidal thickness as early as one month. Such early changes can act as predictive biomarkers of myopia control efficacy over a long term. The utilization of these predictive biomarkers will help clinicians to direct the myopic individuals to appropriate levels of care to control its progression. This has critical implications for the management strategies of ortho-k treatment for myopia control.

## Supplementary Information

**Additional file 1.** Illustration of choroidal vascularity analysis.

**Additional file 2.** IIllustration of choriocapillaris blood perfusion analysis.

**Additional file 3.** Differences in one-month choroidal changes between the fast and slow progression subgroups.

**Additional file 4.** Correlation matrix of choroidal vascularity and choroid thickness.

## Data Availability

The datasets used and/or analyzed during the current study are available from the corresponding author on reasonable request.

## Abbreviations

Ortho-k: Orthokeratology
LA: Luminal area
SA: Stromal area
TCA: total choroidal area
SFCT: Subfoveal choroidal thickness
CVI: Choroidal vascularity index
CcFD: Choriocapillaris flow deficit
OCT: Optical coherence tomography
OCTA: Optical coherence tomography angiography
SS-OCT: Swept-source optical coherence tomography
AL: Axial length
SER: Mean spherical equivalent refraction
RPE: Retinal pigment epithelium
ROC: Receiver operating characteristic curve
AUC: Area under the curve
IDI: Integrated discrimination improvement
