# Supplementary material for: Choroidal vasculature act as predictive biomarkers of long-term ocular elongation in myopic children treated with orthokeratology: a prospective cohort study
Source: Eye Vis (Lond). 2023 Jun 6;10:27. doi: 10.1186/s40662-023-00345-2 (PMC10242233; doi:10.1186/s40662-023-00345-2)

**Additional file 1. Illustration of choroidal vascularity analysis. a** Original OCT image; **b** Automatically segmented choroidal area; **c** Binarized image to differentiate the luminal and stromal areas. **d** Overlay of binarized choroidal area on original image, and the 6 mm submacular area was the region of interest.


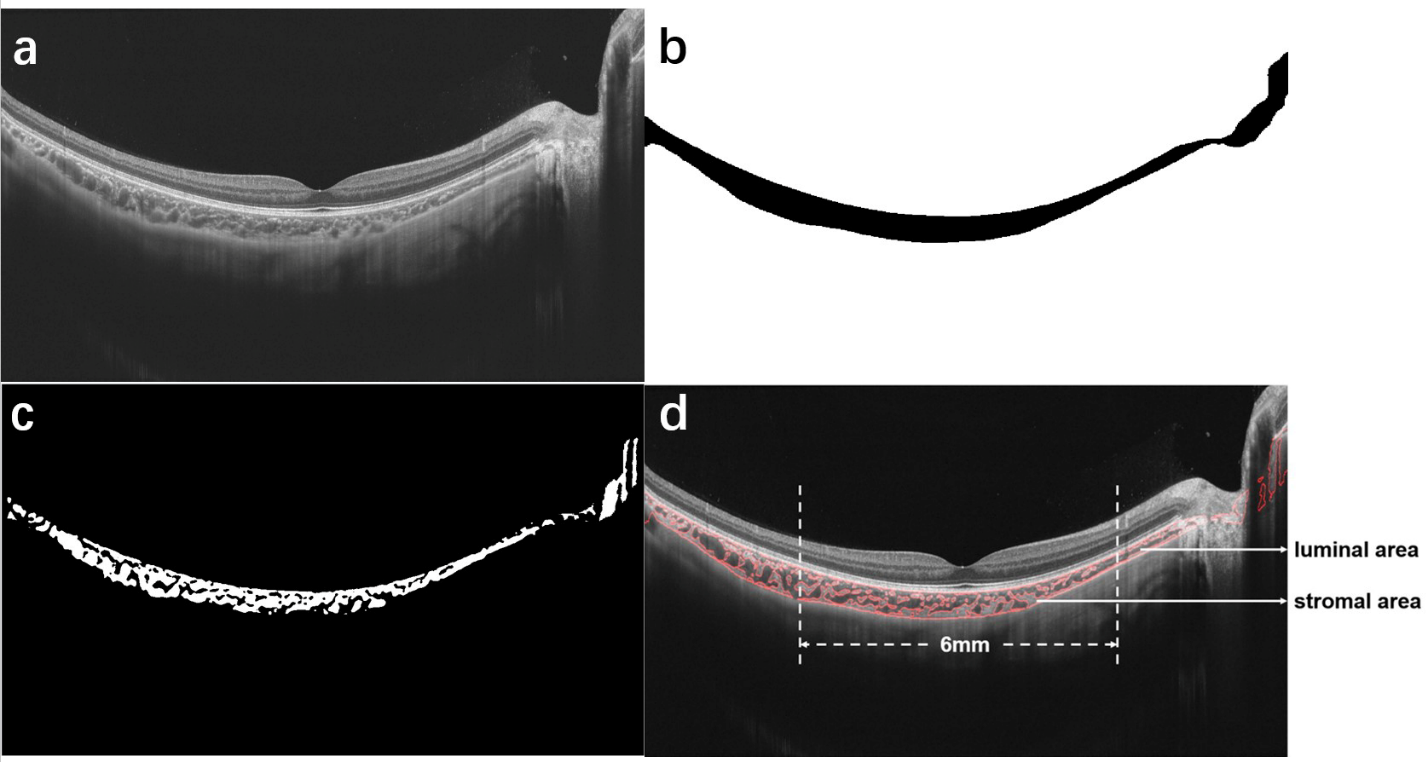

Supplement: Supplementary file 1 — Additional file 1. Illustration of choroidal vascularity analysis. [file 40662_2023_345_MOESM1_ESM.docx]
