# Supplementary material for: Choroidal vasculature act as predictive biomarkers of long-term ocular elongation in myopic children treated with orthokeratology: a prospective cohort study
Source: Eye Vis (Lond). 2023 Jun 6;10:27. doi: 10.1186/s40662-023-00345-2 (PMC10242233; doi:10.1186/s40662-023-00345-2)

**Additional file 2. Illustration of choriocapillaris blood perfusion analysis.** **a** OCTA scan region of 6 × 6 mm; **b** Magnified *en face* OCTA choriocapillaris image; **c** Inverted binarized image, and the 5 mm diameter circular region was the region of interest. OCTA, optical coherence tomography angiography


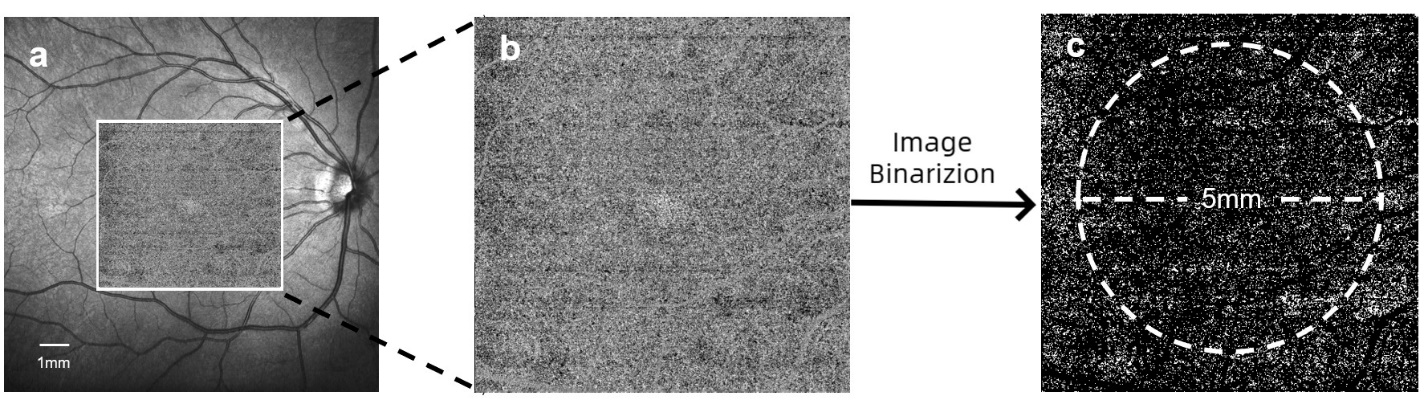

Supplement: Supplementary file 2 — Additional file 2. IIllustration of choriocapillaris blood perfusion analysis. [file 40662_2023_345_MOESM2_ESM.docx]
