# Supplementary material for: Choroidal vasculature act as predictive biomarkers of long-term ocular elongation in myopic children treated with orthokeratology: a prospective cohort study
Source: Eye Vis (Lond). 2023 Jun 6;10:27. doi: 10.1186/s40662-023-00345-2 (PMC10242233; doi:10.1186/s40662-023-00345-2)
Supplement: Supplementary file 3 — Additional file 3. Differences in one-month choroidal changes between the fast and slow progression subgroups. [file 40662_2023_345_MOESM3_ESM.docx]

**Additional file 3. Differences in one-month choroidal changes between the fast and slow progression subgroups.**

| **One-month change** | **Total**  **(n=50)** | **Progression ≤ 0.16 mm (n=25)** | **Progression > 0.16 mm (n=25)** | ***P* value** |
| --- | --- | --- | --- | --- |
| LA (mm^2^) | 0.03±0.07 | 0.06±0.08 | 0.01±0.06 | **0.012** |
| SA (mm^2^) | 0.02±0.05 | 0.04±0.03 | 0.01±0.05 | **0.015** |
| TCA (mm^2^) | 0.06±0.11 | 0.10±0.09 | 0.01±0.10 | **0.005** |
| SFCT (μm) | 10.62±19.98 | 17.33±17.55 | 3.91±20.32 | **0.023** |
| CVI (%) | −0.18±1.90 | −0.27±2.00 | −0.10±1.82 | 0.559 |
| CcFD (%) | 0.19±1.44 | −0.10±1.55 | 0.47±1.30 | 0.185 |

LA = luminal area; SA = stromal area; TCA = total choroidal area; SFCT = subfoveal choroidal thickness; CVI = choroidal vascularity index; CcFD = choriocapillaris flow deficits.

*P* value determined by covariance analysis.

Bold font indicates statistical significance.
