# Supplementary material for: Choroidal vasculature act as predictive biomarkers of long-term ocular elongation in myopic children treated with orthokeratology: a prospective cohort study
Source: Eye Vis (Lond). 2023 Jun 6;10:27. doi: 10.1186/s40662-023-00345-2 (PMC10242233; doi:10.1186/s40662-023-00345-2)

**Additional file 4. Correlation matrix of choroidal vascularity and choroid thickness.** LA, luminal area; SA, stromal area; TCA, total choroidal area; CVI, choroidal vascularity index; SFCT, subfoveal choroidal thickness. X: the *P* value of the correlation was greater than 0.05.


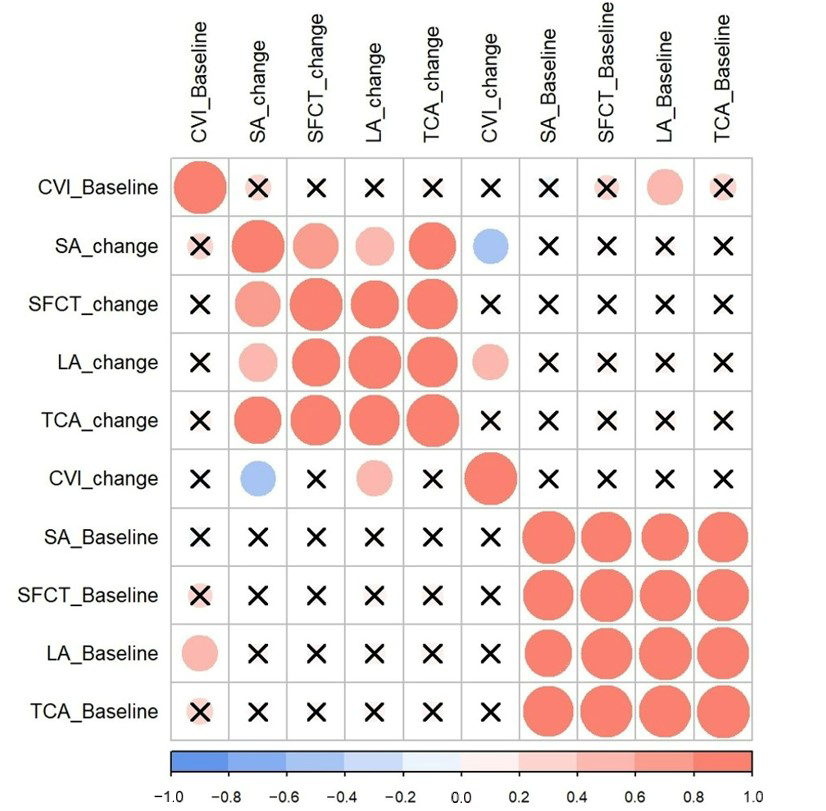

Supplement: Supplementary file 4 — Additional file 4. Correlation matrix of choroidal vascularity and choroid thickness. [file 40662_2023_345_MOESM4_ESM.docx]
